# The impact of experiences of violence on the physical and mental health of a portuguese sample

**DOI:** 10.1192/j.eurpsy.2021.1918

**Published:** 2021-08-13

**Authors:** G. Esgalhado, L. Ferreira

**Affiliations:** Psychology And Education, University of Beira Interior, Covilha, Portugal

**Keywords:** Experiences of Violence, mental health, Physical health

## Abstract

**Introduction:**

Violent Experiences result in economic and social costs for society, impacting on emotions in families, on health (both physical and mental), and overall quality of life, causing potential damages. Thus, it becomes relevant to do research on this impact, aiming at raising awareness and promoting prevention.

**Objectives:**

The purpose of the study is to estimate the impact of experiences of violence on both physical and mental health taking into account variables such as age, gender, and marital status.

**Methods:**

This is a cross-sectional study sampling 1407 Portuguese speaking adults, with an age average of 42 years old (DP=17.28). The measures used were: The SF-36 questionnaire to assess quality of life, physical and mental health, and the Experiences of Violence Questionnaire.

**Results:**

The sample was divided into two groups (victims and non-victims). The group of participants that were not subjected to violence presents more positive results. In relation to the comparison between genders, it was verified that males present more positive results having into account all dimensions SF-36 when compared to women. Also, older participants (53 years old or more) presents lower results of general health.

**Conclusions:**

Violence and health, increasingly related due to the impact it has on the subjects’ physical and mental health and quality of life.

**Disclosure:**

No significant relationships.

